# Validation of the PhageDx™ *Listeria* Assay for Detection of *Listeria* Spp. on Stainless Steel and Ceramic Environmental Surfaces AOAC *Performance Tested Method*^SM^ 102005

**DOI:** 10.1093/jaoacint/qsab036

**Published:** 2021-03-18

**Authors:** Minh M Nguyen, Jose Gil, Wendy Hahn, Thai Dong, John Paulson, Dustin Stevens, Henriett Zahn, Brinna Zimmer, Stephen Erickson

**Affiliations:** 1 Laboratory Corporation of America®/MedTox, 402 County Road D West, St. Paul, MN 55112, USA; 2 Laboratory of America Corporation®/National Genetics Institute, 2440 Sepulveda Blvd., Suite 235, Los Angeles, CA 90064, USA

## Abstract

**Background:**

The PhageDx™ *Listeria* Assay is a simple, specific, and sensitive assay based on the infection of *Listeria* spp. by selected bacteriophages and the resultant expression of a luciferase reporter gene. Results are generated in as little as 24.5 h for stainless steel and ceramic environmental surfaces.

**Objective:**

An AOAC *Performance Tested Methods*^SM^ (PTM) study was conducted to validate the PhageDx *Listeria* Assay for the detection of *Listeria* on stainless steel and ceramic surfaces.

**Method:**

The performance of the PhageDx method was compared to that of the U.S. Food and Drug Administration (FDA) *Bacterial Analytical Manual* (BAM) Ch. 10. Inclusivity/exclusivity, product consistency and stability, and robustness testing also were conducted.

**Results:**

Inclusivity testing demonstrated that the reporter bacteriophages were specific for *Listeria* ssp. and detected 58/61 *Listeria* strains tested, including all 34 *L. monocytogenes* strains. The reporter bacteriophage also was shown to not detect 46/47 non-*Listeria* bacteria in exclusivity testing. Robustness testing showed that the method performed well with specific deviations from the standard protocol. Consistency and stability testing demonstrated that the recombinant phage gave consistent results across three production lots and was stable when stored under appropriate conditions for at least 6 months. Matrix studies on stainless steel and ceramic surfaces showed that there was no significant difference between the PhageDx *Listeria* Assay and the FDA/BAM Chapter 10 reference method.

**Conclusions and Highlights:**

The validation study demonstrates that the PhageDx *Listeria* Assay is an effective method for the detection of *Listeria* spp. on stainless steel and ceramic environmental surfaces and meets the qualifications for AOAC PTM status.

## General Information

*Listeria monocytogenes* is a bacterial pathogen that can be found in moist environments such as soil, water, and decaying vegetation and when ingested, most commonly through eating contaminated food, can cause listeriosis. In the United States, listeriosis is estimated to affect approximately 1600 people resulting in about 260 deaths annually. Symptoms of infection include diarrhea, vomiting, nausea, headaches, stiff neck, confusion, loss of balance, convulsions, fever, and muscle aches. Populations that are particularly susceptible to the effects of listeriosis are pregnant women and their newborns, older adults (65+ years) and people with weakened immunity, where infections can result in death. Infections in pregnant women can also result in miscarriage, stillbirth, premature delivery, or life threatening infant infections (3, 4).

Common food sources of *Listeria* contamination include deli meats, dairy products, and produce. *Listeria spp.* can survive in a wide range of conditions typically used in food preservation such as extremes in temperature and pH and high salt concentrations. Food contamination commonly occurs via transfer from contaminated surfaces that the food comes in contact with (3, 4). Thus, an essential measure to prevent *Listeria* contamination is to monitor sanitation of food preparation areas.

The PhageDx™ *Listeria* Assay provides all the reagents necessary for sample infection, lysis, and luciferase detection. After a 20–24 h enrichment of the test portions in buffered *Listeria* enrichment broth (BLEB) at 35 ± 1°C for stainless steel and ceramic surfaces (4 × 4” test area), a portion of the enriched sample is placed in a well of a 96-well break-away plate and infected with the PhageDx *Listeria* Recombinant Phage at 30°C for 4 h. Then, lysis/luciferase substrate master mix is added to the infected sample and read on a luminometer for signal measurement. Results are expressed as relative light units (RLU). Total sample handling time is approximately 30 min.

## Principle

The PhageDx *Listeria* Assay is a simple, specific, and sensitive diagnostic test for *Listeria* on ceramic and stainless-steel environmental surfaces. The kit is designed to be used with a Promega luminometer and the clear results indicate which samples contain viable *Listeria.* The assay consists of five basic steps; sample collection, enrichment, phage infection, substrate addition, and signal read. Presumptive results are available in 24.5 h which includes a 20–24 h enrichment step and a 4 h infection step. The PhageDx *Listeria* Assay is designed to be performed by qualified laboratory personnel in laboratories performing microbiological analysis.

The PhageDx *Listeria* Assay is based on the infection of *Listeria* spp. by bacteriophages and replication of the infecting bacteriophages within their specific hosts. Bacteriophages demonstrate a high specificity for their bacterial host and are capable of replicating within their host quickly to high numbers. The recombinant phages used in the PhageDx *Listeria* Assay also express a luciferase reporter during replication. The presence of *Listeria* spp*.* is determined by incubating the lysate with the appropriate luciferase substrate and detecting emitted light in a luminometer. An absence of detected light above background indicates that no viable *Listeria* are present in that sample.

## Scope of Method

- *Target organism*.—*Listeria spp*. [*L. monocytogenes* (1/2a, 1/2b, 1/2c, 3a, 4a, 4b, 4c, 4d, 4e), *L. innocua*, *L. ivanovii*, *L. seeligeri*, *L. welshimeri*, *L. grayi*].
- *Matrix*.—Stainless-steel and ceramic surfaces (4 × 4”).
- *Summary of validated performance claims*.—Performance comparable to that of the US Food and Drug Administration (FDA) *Bacterial Analytical Manual* (BAM) Chapter 10, *Detection of Listeria monocytogenes in Foods and Environmental Samples*, and *Enumeration of Listeria monocytogenes in Foods* (1).

## Definitions

- *Probability of detection (POD).*—The proportion of positive analytical outcomes for a qualitative method for a given matrix at a given analyte level or concentration. POD is concentration dependent. Several POD measures can be calculated: POD_R_ (reference method POD), POD_C_ (confirmed candidate method POD), POD_CP_ (candidate method presumptive result POD), and POD_CC_ (candidate method confirmation result POD).
- *Difference of probabilities of detection (dPOD).*—Difference of probabilities of detection is the difference between any two POD values. If the confidence interval of a dPOD does not contain zero, then the difference is statistically significant at the 5% level (2).

## Materials and Methods

### Test Kit Information

- *Kit name*.—PhageDx *Listeria* Assay.
- *Cat. No.*—5013.
- *Ordering information.*—Not applicable. For internal use at Laboratory Corporation of America only.

### Test Kit Components

- *PhageDx Listeria recombinant phage.*—Part No. 3401, 12 tubes containing 100 µL phage solution.
- *Lysis buffer.*—Part No. 3010, 12 tubes containing 150 µL lysis buffer.
- *Assay buffer.*—Part No. 3003, 12 tubes containing 500 µL assay buffer.
- *Luciferase substrate.*—Part No. 3004, 12 tubes containing 10 µL luciferase substrate.
- *96-well break-apart plate.*—Part No. 3005, one pouch containing white break-apart plate (8 wells × 12 strips).
- *Plate sealing tape*.—Part No. 3011, 2 pieces per kit.
- *One package insert.*—Part No. 3402.

### Additional Supplies and Reagents

- *Sample sponge/bag.*—Polyurethane sponge with Letheen, EZ Reach™ Polyurethane Sponge Sampler with Letheen (World Bioproducts, cat. No. EZ18FR-10LET-PUR).
- *Racks for holding sample bag.*
- *Buffered Listeria enrichment broth (BLEB)*.—(Thermo Scientific Remel™ Listeria Enrichment Broth, cat. No. R453692).
- *Sterile filtered tip*s.—For sampling and delivering of 10–1000 µL.
- *Appropriate personal protective equipment.*—*See*https://www.cdc.gov/labs/pdf/CDC-BiosafetyMicrobiologicalBiomedicalLaboratories-2009-P.PDF (5). For confirmation (optional):
- *RAPID’ Listeria plates*.—Bio-Rad RAPID’ *L. mono* Agar, cat. No. 3564293; RAPID’ *L. mono* supplement 1, cat. No. 3564294; RAPID’ *L. mono* supplement 2, Cat. No. 3564746.

### Apparatus

- *Adjustable single channel pipettors.*—For sample collection and delivery (10–1000 µL).
- *Vortex mixer.*
- *Air incubators capable of 30 ± 1°C, 35 ± 1°C, and 37 ± 1°C.*
- *Promega GloMax^®^ 96 or Navigator luminometer.*
- *Personal computer for luminometer control and data analysis.*

### Safety Precautions

- The PhageDx *Listeria* Assay involves the enrichment of samples which may contain human pathogenic *Listeria* and have the potential for contamination with subsequent handling of those samples. This method should be conducted by properly trained laboratory personnel in a suitable microbiology laboratory in accordance with “Biosafety in Microbiological and Biomedical Laboratories,” US Department of Health and Human Services, https://www.cdc.gov/labs/pdf/CDC-BiosafetyMicrobiologicalBiomedicalLaboratories-2009-P.PDF (5). Care should be taken when handling the sample and reagents while performing the method.
- Materials and reagents provided in the PhageDx *Listeria* Assay are not considered hazardous if used according to the assay method. Please review the Material Safety Data Sheet prior to performing the assay.
- Follow all relevant guidelines and laboratory protocols while performing the assay and manufacturer’s equipment instructions.

### General Preparation

- Prepare BLEB media according to manufacturer’s instructions.
- Before using the reagents, flick or spin the tube to collect all of the solution at the bottom of the tube.
- Before adding the pre-warmed BLEB to the sample, confirm that the media and incubator are warmed to 35 ± 1°C. Do not allow the pre-warmed media to cool before adding to the sample. Maintain the media at 35 ± 1°C in an incubator or water bath if preparing multiple samples. It is important to maintain the temperature of the sample and BLEB media used in the enrichment incubation.
- Prepare the Promega luminometer by following the manufacturer’s cleaning procedure and make sure there are no signal “hot spots” that will affect the sample results by reading an empty plate. All signals should be less than 20 RLU. Set up the luminometer to read the appropriate wells on the plate using the pre-programmed PhageDx Assay protocol or, alternatively, set up a read protocol with a 3 min or 180 s delay between starting the program and the start of the signal reads and two plate read runs with a 1 s integration per sample.

### Sample Preparation

- For each surface type, use the EZ Reach Polyurethane Sponge Sampler to sample each test area. Swab environmental surfaces using firm and even pressure vertically (approximately 10 times), then flip the sampler and use the other side to swab horizontally (approximately 10 times) and diagonally (approximately 10 times). Replace sponge back into sample bag and break off and discard handle. Store at room temperature (20–25°C) for 2 h prior to enrichment.
- Add 20 mL of BLEB media pre-warmed to 35 ± 1°C and massage sponge for 10–20 s. Incubate sponge at 35 ± 1°C for 20–24 h.
- Remove the enriched samples from the incubator and massage the sponge in media for 10–20 s. Keeping the sponge in the bag, move sponge away from media and squeeze media from sponge and gently mix media in the bag. *Note*: It is critical that the analyte is released into the media and evenly distributed throughout the sample.
- Immediately after mixing the media, using a single channel pipettor and fresh sterile tip for each sample, transfer 150 µL of the enriched sample into a well of the 96-well plate, one well per sample. *Note:* If continuing to confirmation assay, replace sponge into the media and continue to enrich at 35 ± 1°C for an additional 4–8 h for a total of 24–28 h.
- Remove one tube containing the recombinant phage solution for each eight well strip used. Flick or spin the tube to collect all of the solution at the bottom of the tube. Add 10 µL of recombinant phage to the 150 µL sample and mix thoroughly by gently pipetting up and down, being careful not to introduce bubbles. Cover samples with plate sealing tape to prevent evaporation and cross contamination and place the 96-well plate in the 30 ± 1°C incubator for 4 h.
- Remove one tube containing the lysis buffer, assay buffer, and substrate for each eight well strip used and thaw to room temperature. Flick or spin the tubes to collect all of the solution at the bottom of the tubes. Prepare the lysis/luciferase master mix by transferring the entire contents of assay buffer (0.5 mL) and lysis buffer (150 µL) tubes to the substrate tube (10 µL) and mix well by either vortexing or inverting the tube several times. *Note:* Use within 1 h of preparation.
- Using a clean tip for each sample, add 65 µL of the lysis/luciferase master mix to each well using a single channel pipettor. Mix thoroughly by pipetting up and down, being careful not to introduce bubbles.
- Once all of the samples have received the lysis/luciferase master mix, place the sample plate in the luminometer, close the lid, and initiate the read program.

### Interpretation and Test Result Report

- The luminometer program will display the results on the screen as RLU values corresponding to the well positions of the break-apart plate.
- Samples positive for *Listeria* will have a reading value of 300 RLU or greater. Negative samples will be less than 300 RLU.
- Once all of the samples have been run and analyzed, remove the plate from the luminometer and follow the manufacturer’s instructions for cleaning the instrument and shut down. *Note:* In some cases, the PhageDx *Listeria* Assay will generate a very high signal and result in adjacent wells reading as a false positive due to a bleed-over signal from a well with a high signal. If a sample well is positive and has a 1000× lower signal than the adjacent sample well with a higher signal (typically in the range of 300–2000 RLU), this could be a result of bleed-over signal. In these cases, we recommend that the contents of the potential false positive well (lower RLU sample) be transferred to a new well at least a 2–3 well distance from the high signal well or to a new strip and re-read to confirm that the signal is from the sample and not a result of bleed-over signal.

### Confirmation

We recommend that presumptive positives from the phage assay be confirmed.

- To confirm, continue to enrich sponge samples for an additional 4–8 h for a total of 24–28 h at 35 ± 1°C. This is/can be done concurrently with the 4 h phage infection step. Sponge samples that were positive with the phage assay can then proceed to the next steps.
- Massage sponge and contents of sample bag vigorously for 10–20 s to mix contents well. Remove a 100 µL aliquot and streak onto RAPID’ *Listeria* plates (Bio-Rad RAPID’ *L. mono* Agar, cat. No. 3564293; RAPID’ *L. mono* supplement 1, cat. No. 3564294; RAPID’ *L. mono* supplement 2, cat. No. 3564746). Incubate for an additional 24–48 h at 37 ± 1°C. *L. monocytogenes* forms blue (pale blue, grey-blue, or dark blue) colonies. *L. ivanovii* forms blue-green colonies with a yellow halo. Other *Listeria* spp. form white colonies with or without a yellow halo. Refer to manufacturer’s product insert for detailed description.
- Confirm plate test results by testing individual colonies using approved qPCR tests. Alternatively, the user may use another approved reference method confirmation procedure.

## Validation Study

This validation study was conducted under the AOAC Research Institute *Performance Tested Method(s)*^SM^ (PTM) program and the *AOAC INTERNATIONAL Methods Committee Guidelines for Validation of Microbiological Methods for Food and Environmental Surfaces, Appendix J* (6). Method developer studies were conducted in the laboratories of Laboratory Corporation of America Holdings, and included the inclusivity/exclusivity study, product consistency and stability studies, and robustness testing. The independent laboratory study was conducted by Q Laboratories, Inc., and included inclusivity and exclusivity studies for selected strains and matrix studies for all claim matrixes.

### Method Developer Studies

#### (a) Inclusivity and exclusivity studies.—

Inclusivity (*Listeria*) and exclusivity strains were obtained from academic, governmental, and commercially available sources. A total of 61 *Listeria* strains were tested for the inclusivity study, 54 strains by LabCorp and an additional seven by Q Laboratories. For inclusivity testing done at LabCorp, 54 *Listeria* species covering *L. monocytogenes* serotypes 1/2a, 1/2b, 1/2c, 3a, 4a, 4b, 4c, 4d, and 4e, *L. innocua*, *L. ivanovii*, *L. seeligeri*, *L. welshimeri*, and *L*. *grayi* strains were examined. Each strain was grown at 35 ± 1°C for 20–24 h in BLEB medium. The cultures were then diluted to approximately 100 times the LOD_50_ of the PhageDx *Listeria* Assay and transferred to a 96-well plate.

A total of 47 non-*Listeria* strains were tested for the exclusivity study, 45 strains by LabCorp and three by Q Laboratories (*Enterococcus faecalis* ATCC 29212 tested by both LabCorp and Q Laboratories). For exclusivity testing done at LabCorp, 45 different strains of non-*Listeria* species and strains were grown under optimal enrichment conditions suitable to the strain type (medium and temperature) for a minimum of 20–24 h. One hundred microliters of the undiluted cultures were transferred to 96-well plates.

For both inclusivity and exclusivity studies, samples were infected with 10 µL of PhageDx *Listeria* phage reagent at 30 ± 1°C for 4 h. After infection, 65 µL of lysis/luciferase master mix was added to the wells and samples were read on a luminometer. Samples with a signal of ≥300 RLU were considered positive.

#### (b) Product consistency (lot-to-lot) and stability studies.—

Three separate production lots of PhageDx *Listeria* recombinant phage were prepared according to written manufacturing documents and tested according to quality control procedures. Quality control procedures verified that each lot when diluted to working concentration had the same titer, background, and level of detection. Recombinant phage reagents were aged between 0 and 6 months when assayed for stability.

Consistency and stability were done according to AOAC guidance, where a sample was inoculated with *L. monocytogenes* (ATCC 7302) to give fractional positives. Ten replicates were run in the PhageDx Assay, and the RLU values analyzed. A set of stability studies was also conducted using the non-target bacterium *Enterococcus faecalis (ATCC 29212).* Overnight cultures of *E. faecalis* were used directly in the assay.

#### (c) Robustness study.—

Three parameters were varied to demonstrate assay robustness: infection time (±30 m), recombinant phage concentration (±20%), and lysis/luciferase master mix amount (±5 µL). Briefly, stainless-steel surfaces were inoculated with *L. monocytogenes* (ATCC 19115) at a low level to generate partial positives or 100× *E. faecalis* (ATCC 29212) and allowed to dry and sit for 16–24 h at room temperature (20–25°C). The PhageDx *Listeria* Assay protocol was followed with the variations in infection time, recombinant phage concentration, and lysis/substrate master mix amounts as indicated in Table 4. Samples with RLU values of 300 or greater were considered positive. Samples were confirmed by allowing samples to enrich for a total of 24–28 h and plating on RAPID’ *L. monocytogenes* plates and incubating at 37 ± 1°C for an additional 24–48 h. The presence of blue (pale blue, grey-blue, or dark blue) colonies on selective plates indicated a positive result for *L. monocytogenes*.

### Independent Laboratory Validation Study

#### (a) Inclusivity and exclusivity.—

The inclusivity and exclusivity study evaluated two strains of *L. grayi*, three strains of *L. seeligeri*, two strains of *L. welshimeri*, one strain of *Enterococcus faecalis*, one strain of *Enterococcus faecium*, and one strain of *Streptococcus pyogenes*. All cultures evaluated were propagated from a stock culture stored at −70°C to tryptic soy agar (TSA) with 5% sheep blood agar (SBA) and incubated for 24 ± 2 h at 35 ± 1°C. After incubation of the target organisms, a single colony from SBA was transferred to a 9 mL aliquot of BLEB and incubated for 20–24 h at 35 ± 1°C. Exclusivity strains were transferred to non-selective media and incubated under conditions for optimal growth. Inclusivity and exclusivity testing was done in the same manner as method developer’s inclusivity and exclusivity studies. All inclusivity and exclusivity strains were blind coded and randomized. Tests results were decoded and reported as either positive or negative.

#### (b) Matrix study.—

The independent laboratory evaluation included matrix studies for 4 × 4” test areas of stainless steel (18 GA 300 series, brush finish, NSF certified stainless steel) and ceramic (unglazed) surfaces, comparing the PhageDx *Listeria* Assay to the 2017 FDA/BAM Chapter 10. Within each sample set, there were five uninoculated samples (0 CFU/test portion), 20 low-level inoculated samples (0.2–2 CFU/test portion), and five high-level inoculated samples (2–10 CFU/test portion). The low inoculation level was designed to produce fractional positive results in which the candidate or reference method produced 5–15 positive results (25–75%).

All samples were analyzed by the PhageDx *Listeria* Assay following enrichment with pre-warmed (35 ± 1°C) BLEB and incubated for 20–24 h at 35 ± 1°C. Analysis was conducted after 20 h of enrichment. Regardless of presumptive results, all samples were culturally confirmed by the FDA/BAM Chapter 10 reference method. In addition, all samples were confirmed following the alternative confirmation as described in *Confirmation*. Final confirmation for all samples was obtained by Bruker MALDI Biotyper following AOAC Method **2017.10** (7).

##### (c) Organism preparation and inoculation.—

For ceramic surfaces, 4 × 4” areas were inoculated with 0.25 mL of diluted *L. innocua* (ATCC 43547) culture and sampled using sampling sponges pre-moistened in letheen broth. For stainless steel surfaces, 4 × 4” areas were inoculated with 0.25 mL of diluted *L. monocytogenes* 4a (CWD 1554). In addition to the *L. monocytogenes* culture, stainless-steel surface was co-inoculated with a competitor organism, *Enterococcus faecalis* (ATCC 29212), at 10× the level of the target organism. All cultures utilized were propagated from a stock culture stored at −70°C to SBA and incubated for 24 ± 2 h at 35 ± 1°C. After incubation, a single colony from SBA was transferred to a 9 mL aliquot of brain heart infusion (BHI) broth for 20–24 h at 35 ± 1°C. Each culture was then diluted to the target concentration by performing serial dilutions using 0.1% peptone water as the diluent. For the uninoculated test portions, sterile 0.1% peptone water was applied to the test area. Each surface was dried for 16–24 h at room temperature (24 ± 2°C) prior to sampling. To determine the inoculation level for the environmental surfaces, aliquots of each inoculum were plated onto TSA in triplicate.

##### (d) FDA/BAM Chapter 10.—

For environmental samples, sponges were pre-moistened with 10 mL of Dey-Engley (D/E) neutralizing broth. Following addition of D/E neutralizing broth surfaces were swabbed using firm and even pressure 10 times vertically and horizontally. All environmental samples were then stored at 22 ± 2°C for 2 h ± 15 min. Swabs were enriched with 90 mL of BLEB, massaged by hand for 2 min and incubated at 30 ± 1°C for 4 h. Following 4 h of incubation, selective supplements acriflavine (10 mg/L), sodium nalidixate (40 mg/L), and cycloheximide (50 mg/L) were added to each test portion and samples were re-incubated for 24–48 h.

After 24 h of total incubation, the enriched samples were streaked to modified Oxford (MOX) agar plates and incubated at 35 ± 1°C for 24–48 h. In addition to MOX each enriched sample was streaked to the chromogenic selective agar brilliance *Listeria* agar (BLA) and incubated at 37 ± 1°C for 24 ± 2 h. The enriched samples were re-incubated for an additional 24 h at 30 ± 1°C and then streaked to a second MOX and BLA agar plate which was incubated for 24–48 h at 35 ± 1°C and 37 ± 1°C for 24 ± 2 h, respectively. MOX and BLA agar plates were examined for suspect colonies, and if present, at least five colonies were streaked to TSA containing 0.6% yeast extract (TSA/YE). The TSA/YE plates were incubated at 35 ± 1°C for 24–48 h and then examined for purity. Pure colonies were tested for catalase reactivity and a Gram stain was conducted. A pure *L. monocytogenes* colony was transferred to trypticase soy broth containing 0.6% yeast extract (TSB/YE). The TSB/YE cultures were incubated at 25 ± 1°C overnight, or until the broth was turbid, indicating sufficient growth. Catalase-positive organisms were stabbed into SBA plates and incubated at 35 ± 1°C for 24–48 h. The TSB/YE tubes incubated at 25 ± 1°C were used to prepare a wet mount slide to determine motility pattern. After incubation, the SBA plates were examined for hemolysis. Final confirmation was conducted using Bruker MALDI Biotyper following AOAC Method **2017.10**.

##### (e) PhageDx Listeria Assay.—

Sponges from ceramic (4 × 4” test area) and stainless steel (4 × 4” test area) swabbed surfaces were enriched according to the protocol described in *Organism preparation and inoculation*.

PhageDx *Listeria* Assay was performed as described in *Sample preparation*. Briefly, using a polyurethane sponge pre-moistened with letheen, surfaces were swabbed and the sponge placed into the sample bag and held at 22 ± 2°C for 2 h ± 15 m. Swabs were enriched with 20 mL of pre-warmed BLEB, massaged by hand for 10–20 s and incubated at 35 ± 1°C for 20 h. Following enrichment, 150 µL sample aliquots were transferred to a 96-well plate. Ten microliters of phage reagent was added to each sample and samples were incubated at 30 ± 1°C for 4 h. After infection, 65 µL of lysis/luciferase substrate master mix was added to the samples and then read on a luminometer. All samples were culturally confirmed by the FDA/BAM Chapter 10 reference method. All samples were also confirmed by an alternate confirmation described previously in *Confirmation*. Final confirmation for all samples was obtained by Bruker MALDI Biotyper following AOAC Method **2017.09**.

## Results

Inclusivity studies for the PhageDx Listeria Assay demonstrated that of the 61 *Listeria* inclusivity strains tested, 58 were detected and three were not detected (Table 1). All 34 *L. monocytogenes* strains tested were detected, while those strains that were not detected included one strain each of *L. innocua* (ATCC BAA-680), *L. seeligeri* (ATCC 51335), and *L. welshimeri* (ATCC 43551; Table 1). Those strains that were not detected can be divided into two categories. The first category includes those that would be detected with higher cell numbers. *L. innocua* (ATCC BAA-680) and *L. welshimeri* (ATCC 43551) were detected with 20 h enrichment, or within the recommended enrichment time frame for environmental samples (data not shown). *L. seeligeri* (ATCC 51335), however, remained negative with extended enrichment times.

Exclusivity studies showed that of the 47 exclusivity strains examined, 46 of the 47 strains were negative (Table 2). One strain, *Lactobacillus plantarum* ATCC 14917, was reported as positive; however, the signal generated was significantly lower than that of *Listeria* strains (data not shown).

**Table 1.** PhageDx *Listeria* inclusivity list

No.	Species	Serogroup^a^	Source	Origin	PhageDx Result
1	*L. monocytogenes*	1/2a	ATCC 15313^b^	Rabbit	Positive
2	*L. monocytogenes*	1/2a	ATCC 51774	Human	Positive
3	*L. monocytogenes*	1/2a	ATCC 35152	Guinea Pig	Positive
4	*L. monocytogenes*	1/2a	ATCC BAA-679	Rabbit	Positive
5	*L. monocytogenes*	1/2a	ATCC 51775	Cheese	Positive
6	*L. monocytogenes*	1/2a	ATCC 19111	Poultry	Positive
7	*L. monocytogenes*	1/2a	ATCC 51772	Cheese	Positive
8	*L. monocytogenes*	1/2b	ATCC BAA-751	Not available	Positive
9	*L. monocytogenes*	1/2b	ATCC BAA-839	Not available	Positive
10	*L. monocytogenes*	1/2b	ATCC 51780	Cheese	Positive
11	*L. monocytogenes*	1/2c	ATCC 51779	Cheese	Positive
12	*L. monocytogenes*	2	ATCC 9525	Not available	Positive
13	*L. monocytogenes*	1/2C	ATCC 19112	Human	Positive
14	*L. monocytogenes*	3a	ATCC 19113	Human	Positive
15	*L. monocytogenes*	3a	ATCC 51782	Cheese	Positive
16	*L. monocytogenes*	4	ATCC 51781	Not available	Positive
17	*L. monocytogenes*	4a	ATCC 19114	Sheep	Positive
18	*L. monocytogenes*	4b	ATCC 19115	Human	Positive
19	*L. monocytogenes*	4b	Cornell FSL-J1-225^c^	Not available	Positive
20	*L. monocytogenes*	4b	ATCC 51776	Cheese	Positive
21	*L. monocytogenes*	4b	ATCC 51778	Cheese	Positive
22	*L. monocytogenes*	4b	ATCC 13932	Human	Positive
23	*L. monocytogenes*	4b	ATCC 51777	Cheese	Positive
24	*L. monocytogenes*	4c	ATCC 19116	Chicken	Positive
25	*L. monocytogenes*	4d	ATCC 19117	Sheep	Positive
26	*L. monocytogenes*	4e	ATCC 19118	Chicken	Positive
27	*L. monocytogenes*		ATCC 700402	Not available	Positive
28	*L. monocytogenes*		ATCC 7302	Not available	Positive
29	*L. monocytogenes*		ATCC 7644	Not available	Positive
30	*L. monocytogenes*		ATCC 23074	Not available	Positive
31	*L. monocytogenes*		ATCC 23073	Not available	Positive
32	*L. monocytogenes*		ATCC 43256	Not available	Positive
33	*L. monocytogenes*		ATCC 984	Not available	Positive
34	*L. monocytogenes*	4a	UVM CWD 1554^d^	Not available	Positive
35	*L. grayi*		ATCC 19120	Not available	Positive
36	*L. grayi*		ATCC 25401	Not available	Positive
37	*L. grayi*		ATCC 700545	Not available	Positive
38	*L.* *grayi*^e^		QL 30911.12^f^	Environmental sample	Positive
39	*L. grayi serovar* *Murrayi*^e^		NCTC10814^g^	Not available	Positive
40	*L. innocua*	6a	ATCC 33090	Not available	Positive
41	*L. innocua*		ATCC 51742	Not available	Positive
42	*L. innocua*	6b	ATCC 33091	Not available	Positive
43	*L. innocua*	6b	ATCC 43547	Not available	Positive
44	*L. innocua*	6a	ATCC BAA-680	Not available	Negative^h^
45	*L. ivanovii*		ATCC 19119	Not available	Positive
46	*L. ivanovii*		ATCC 49953	Not available	Positive
47	*L. ivanovii*	5	ATCC BAA-678	Not available	Positive
48	*L. ivanovii*		ATCC BAA-753	Not available	Positive
49	*L. ivanovii*		ATCC 49954	Not available	Positive
50	*L. seeligeri*		ATCC 35967	Not available	Positive
51	*L. seeligeri*		ATCC 51334	Not available	Positive
52	*L. seeligeri*	4a	ATCC 51335	Not available	Negative
53	*L.* *seeligeri*^e^		QL 031011.2	Creamer	Positive
54	*L.* *seeligeri*^e^		QL 031011.5	Frozen vegetables	Positive
55	*L.* *seeligeri*^e^		Cornell FSL-S4-035	Not available	Positive
56	*L. welshimeri*	6b	ATCC 35897	Not available	Positive
57	*L. welshimeri*	6a	ATCC 43551	Not available	Negative^h^
58	*L. welshimeri*	6a	ATCC 43549	Not available	Positive
59	*L. welshimeri*	1/2b	ATCC 43550	Not available	Positive
60	*L.* *welshimeri*^e^		QL 030911-(0).8	Beef	Positive
61	*L.* *welshimeri*^e^		UVM LW001	Not available	Positive

a Serogroup listed if applicable.

b American Type Culture Collection, Manassas, VA.

c Cornell University, Ithaca, NY.

d University of Vermont, Burlington, VA.

e Testing done by Q Laboratories.

f Q Laboratories, Cincinnati, OH.

g National Collection of Type Cultures, Proton Down, Salisbury, UK.

h 20 h enrichment generated positive result.

Lot-to-lot studies showed that the PhageDx *Listeria* recombinant phages can be manufactured consistently and are stable for at least 6 months when stored at 4°C. Manufactured lots were made on 2/20/20, 4/20/20, and 8/20/20 according to written manufacturing documents. Each lot produced similar results when tested according to QC tests for bacteriophage concentration, background signal, and LOD. Performance tests to determine the stability of each lot were performed to determine the shelf life of the recombinant phage. These tests demonstrated that lots produced 0 months prior to testing showed no significant difference from lots produced at least 6 months prior to testing. Additionally, no variation in exclusivity was observed with these three recombinant phage lots in tests with *E. faecalis*. Results shown in Table 3.

**Table 2.** PhageDx *Listeria* exclusivity list

No.	Species	Source	PhageDx result
1	*Acinetobacter baumannii*	ATCC 19606^a^	Negative
2	*A.* *calcoaceticus*	ATCC 23055	Negative
3	*Bacillus cereus*	ATCC 14579	Negative
4	*B.* *cereus*	ATCC 13061	Negative
5	*B.* *circulans*	ATCC 61	Negative
6	*B.* *coagulans*	ATCC 7050	Negative
7	*B.* *licheniformis*	ATCC 9789	Negative
8	*B.* *megaterium*	ATCC 14581	Negative
9	*B.* *mycoides*	ATCC 6462	Negative
10	*B.* *pumilus*	ATCC 700814	Negative
11	*B.* *subtilis*	ATCC 23857	Negative
12	*B.* *subtilis subsp. subtilis*	ATCC 6051	Negative
13	*B.* *weihenstephanensis*	ATCC 12826	Negative
14	*Citrobacter braakii*	ATCC 51113	Negative
15	*C.* *freundii*	ATCC 8090	Negative
16	*C.* *koseri*	ATCC 25408	Negative
17	*Cronobacter muytjensii*	ATCC 51329	Negative
18	*C.* *sakazakii*	ATCC 12868	Negative
19	*Escherichia coli*	ATCC 9637	Negative
20	*Edwardsiella tarda*	ATCC 15947	Negative
21	*Enterobacter aerogenes*	ATCC 13048	Negative
22	*E.* *cloacae, subsp cloacae*	ATCC 13047	Negative
23	*E.* *kobei*	ATCC BAA-260	Negative
24	*Enteroccus faecalis*	ATCC 19433	Negative
25	*E.* *faecalis*^b^	ATCC 29212	Negative
26	*E.* *faecium*	ATCC 19434	Negative
27	*E.* *faecium*^c^	ATCC 700221	Negative
28	*Escherichia fergusonii*	ATCC 35469	Negative
29	*E.* *hermanni*	ATCC 33650	Negative
30	*Hafnia alevi*	ATCC 13337	Negative
31	*Klebsiella oxytoca*	ATCC 43165	Negative
32	*K.* *pneumonia*	ATCC 4352	Negative
33	*Lactobacillus plantarum*	ATCC 14917	Positive
34	*L.* *rhamnosus*	ATCC 7469	Negative
35	*Morganella morganii: subsp. Maorganii M11*	ATCC 25830	Negative
36	*Pluralibacter gergoviae*	ATCC 33028	Negative
37	*Proteus mirabilis*	ATCC 43071	Negative
38	*P.* *vulgaris*	ATCC 33420	Negative
39	*Pseudomonas aeruginosa; Strain Boston 41401*	ATCC 27853	Negative
40	*Serratia marcescens*	ATCC 13880	Negative
41	*Shigella flexneri*	ATCC 12022	Negative
42	*S.* *sonnei*	ATCC 9290	Negative
43	*Staphylococcus aureus*	ATCC 27660	Negative
44	*S.* *epidermidis*	ATCC 14990	Negative
45	*S.* *haemolyticus*	ATCC 29970	Negative
46	*Streptococcus* *pyogenes*^c^	ATCC 19615	Negative
47	*Yersinia enterocolitica*	ATCC 23715	Negative

a American Typle Culture Collection, Manassas, VA.

b Testing done by both LabCorp and Q Laboratories.

c Testing done by Q Laboratories, Cincinnati, OH.

**Table 3.** Stability and consistency (lot-to-lot) of PhageDx *Listeria* recombinant phage—POD comparison

Phage lot No.	Lot age, months	N^a^	x^b^	POD_A_^c^	95% CI	Phage lot No.	Lot age, months	N	x	POD_B_^d^	95% CI	dPOD_AB_^e^	95% CI^f^
*L. monocytogenes* (target)													
B^g^	4	10	4	0.4	0.17, 0.69	C^h^	0	10	6	0.6	0.31, 0.83	−0.20	−0.53, 0.21
A^i^	6	10	4	0.4	0.17, 0.69	C	0	10	6	0.6	0.31, 0.83	−0.20	−0.53, 0.21
A	6	10	4	0.4	0.17, 0.69	B	4	10	4	0.4	0.17, 0.69	0.00	−0.37, 0.37
*Enterococcus faecalis* (non-target)													
B	4	10	0	0.0	0.0, 0.28	C	0	10	0	0.0	0.0, 0.28	0.0	−0.28, 0.28
A	6	10	0	0.0	0.0, 0.28	C	0	10	0	0.0	0.0, 0.28	0.0	−0.28, 0.28
A	6	10	0	0.0	0.0, 0.28	B	4	10	0	0.0	0.0, 0.28	0.0	−0.28, 0.28

a N = Number of test portions.

b x = Number of positive test portions.

c POD_A_ = Positive outcomes divided by the total number of trials first member of pair.

d POD_B_ = Positive outcomes divided by the total number of trials second member of pair.

e dPOD_AB_ = Difference in POD between the paired comparison.

f 95% CI = If the confidence interval of a dPOD does not contain zero, then the difference is statistically significant at the 5% level.

g Lot B was produced 04/20/20.

h Lot C was produced 08/20/20.

i Lot A was produced 02/20/20.

Robustness testing of the PhageDx *Listeria* Assay demonstrated that variations in infection time, recombinant phage concentration, and lysis/luciferase master mix amounts do not alter the results compared to the standard protocol. Infection times of 3.5 and 4.5 h, recombinant phage volumes of 8 and 12 µL, and lysis/luciferase master mix volumes of 60 and 70 µL produced identical results to the standard protocol of 4 h infection, 10 µL of recombinant phage, and 65 µL of lysis/luciferase master mix in both uninoculated and low inoculum test samples (Table 4). These results indicate that these deviations from the PhageDx *Listeria* Assay protocol did not alter the final results.

**Table 4.** Robustness study: impact of varying infection time, phage concentration, lysis/luciferase master mix concentration on PhageDx *Listeria* Assay results—POD comparison

Test condition^a^	Test parameters			N^c^	Test condition results				Nominal condition results^b^			dPOD_TN_^g^	95% CI^h^
	Infection time, h	Volume phage, µL	Volume substrate, µL		x^d^	${\text{POD}}_{T}^{e}$	95% CI		x	POD_N_^f^	95% CI		
Stainless steel surface—inoculated with *L. monocytogenes* ATCC 19115 (target) 55 CFU/test area													
1	3.5	8	60	10	4	0.40	0.17, 0.69		4	0.40	0.17, 0.69	0.0	−0.25, 0.25
2	3.5	8	70	10	4	0.40	0.17, 0.69		4	0.40	0.17, 0.69	0.0	−0.25, 0.25
3	3.5	12	60	10	4	0.40	0.17, 0.69		4	0.40	0.17, 0.69	0.0	−0.25, 0.25
4	3.5	12	70	10	4	0.40	0.17, 0.69		4	0.40	0.17, 0.69	0.0	−0.25, 0.25
5	4.5	8	60	10	4	0.40	0.17, 0.69		4	0.40	0.17, 0.69	0.0	−0.25, 0.25
6	4.5	8	70	10	4	0.40	0.17, 0.69		4	0.40	0.17, 0.69	0.0	−0.25, 0.25
7	4.5	12	60	10	4	0.40	0.17, 0.69		4	0.40	0.17, 0.69	0.0	−0.25, 0.25
8	4.5	12	70	10	4	0.40	0.17, 0.69		4	0.40	0.17, 0.69	0.0	−0.25, 0.25
Stainless steel surface—inoculated with *E. faecalis* ATCC 29212 (non-target) 4950 CFU/test area													
1	3.5	8	60	10	0	0.0	0.00, 0.28		0	0.0	0.00, 0.28	0.0	−0.25, 0.25
2	3.5	8	70	10	0	0.0	0.00, 0.28		0	0.0	0.00, 0.28	0.0	−0.25, 0.25
3	3.5	12	60	10	0	0.0	0.00, 0.28		0	0.0	0.00, 0.28	0.0	−0.25, 0.25
4	3.5	12	70	10	0	0.0	0.00, 0.28		0	0.0	0.00, 0.28	0.0	−0.25, 0.25
5	4.5	8	60	10	0	0.0	0.00, 0.28		0	0.0	0.00, 0.28	0.0	−0.25, 0.25
6	4.5	8	70	10	0	0.0	0.00, 0.28		0	0.0	0.00, 0.28	0.0	−0.25, 0.25
7	4.5	12	60	10	0	0.0	0.00, 0.28		0	0.0	0.00, 0.28	0.0	−0.25, 0.25
8	4.5	12	70	10	0	0.0	0.00, 0.28		0	0.0	0.00, 0.28	0.0	−0.25, 0.25

a Each test condition is being compared to the nominal test condition.

b Nominal condition = 4 h infection, 10 µL phage, 65 µL lysis/luciferase master mix substrate.

c N = Number of test portions per condition.

d x = Number of positive test portions per condition.

e POD_T_ = Positive outcomes divided by the total number of trials per condition.

f POD_N_ = Positive outcomes divided by the total number of trials per nominal condition.

g dPOD_TN_ = Difference in POD between the test condition and nominal condition.

h 95% CI = If the confidence interval of a dPOD does not contain zero, then the difference is statistically significant at the 5% level.

Matrixes studies were performed as per criteria outlined in Appendix J of the Official Methods of Analysis Manual, and fractional positive results were obtained for the PhageDx Listeria Assay (6). For ceramic and stainless steel (4×4” test areas) environmental samples, the inoculum recovered for the target *L. innocua* and *L. monocytogenes* strains fell within the targeted levels, while the competitor organism was at a level at least 10 times higher. The probability of detection (POD) was calculated as the number of positive outcomes divided by the total number of trials (6). Comparisons of the PhageDx presumptive results to the confirmed results using the FDA/BAM Chapter 10 reference method showed no significant difference between the two (Table 5). No significant difference was found when comparing the results of the PhageDx presumptive results and FDA/BAM Chapter 10 results in an unpaired study (Table 6). There was also no significant difference in the PhageDx presumptive results and results of the PhageDx confirmation method (Table 7). Finally, there was no difference between the presumptive PhageDx results confirmed using the PhageDx confirmation method and the FDA/BAM reference method (Table 8). In summary, POD analysis between the PhageDx Listeria Assay method and the reference method indicated that there was no significant difference at the 5% level between the number of positive results by the two methods for both matrixes. The POD analysis between the PhageDx Listeria Assay presumptive and confirmed results indicated that there was no significant difference at the 5% level for the method for both matrixes. A summary of POD analyses (8) are presented in Tables 5–8.

**Table 5.** PhageDx *Listeria* results: presumptive vs confirmed using the reference method procedure

Matrix	Strain	CFU/test area	N^a^	PhageDx *Listeria* presumptive			BAM Ch. 10 confirmed			${\text{dPOD}}_{\text{CP}}^{e}$	95% CI^f^
				x^b^	${\text{POD}}_{\text{CP}}^{c}$	95% CI	x	${\text{POD}}_{\text{CC}}^{d}$	95% CI		
Stainless steel (4 × 4”)	*L. monocytogenes* 4a (CWD 1554)/*E. faecalis* ATCC 29212^g^^,h^	N/A^i^	5	0	0.00	0.00, 0.43	0	0.00	0.00, 0.43	0.00	−0.47, 0.47
		48 & 510	20	8	0.40	0.22, 0.61	8	0.40	0.22, 0.61	0.00	−0.13, 0.13
		160 & 1800	5	5	1.00	0.57, 1.00	5	1.00	0.57, 1.00	0.00	−0.47, 0.47
Ceramic (4 × 4”)	*L. innocua* (ATCC 43547 6 b)	N/A	5	0	0.00	0.00, 0.43	0	0.00	0.00, 0.43	0.00	−0.47, 0.47
		60	20	9	0.45	0.26, 0.66	9	0.45	0.26, 0.66	0.00	−0.13, 0.13
		200	5	5	1.00	0.57, 1.00	5	1.00	0.57, 1.00	0.00	−0.47, 0.47

a N = Number of test portions.

b x = Number of positive test portions.

c POD_CP_ = Candidate method presumptive positive outcomes divided by the total number of trials.

d POD_CC_ = Candidate method confirmed positive outcomes (confirmed using the reference method procedure) divided by the total number of trials.

e dPOD_CP_ = Difference between the candidate method presumptive result and candidate method confirmed result POD values.

f 95% CI = If the confidence interval of a dPOD does not contain zero, then the difference is statistically significant at the 5% level.

g CWD = University of Vermont, Burlington, VT.

h ATCC = American Type Culture Collection, Manassas, VA.

i N/A = Not applicable.

**Table 6.** Method comparison results: PhageDx *Listeria* vs BAM Ch. 10

Matrix	Strain	CFU/test area	N^a^	PhageDx *Listeria* results			BAM Ch. 10			${\text{dPOD}}_{\text{C}}^{e}$	95% CI^f^
				x^b^	${\text{POD}}_{\text{C}}^{c}$	95% CI	x	${\text{POD}}_{\text{R}}^{d}$	95% CI		
Stainless steel (4 × 4”)	*L. monocytogenes* 4a (CWD 1554)/E. faecalis ATCC 29212^g^^,h^	N/A^i^	5	0	0.00	0.00, 0.43	0	0.00	0.00, 0.43	0.00	−0.43, 0.43
		48 & 510	20	8	0.40	0.22, 0.61	8	0.40	0.22, 0.61	0.00	−0.28, 0.28
		160 & 1800	5	5	1.00	0.57, 1.00	5	1.00	0.57, 1.00	0.00	−0.43, 0.43
Ceramic (4 × 4”)	*L. innocua* (ATCC 43547 6 b)	N/A	5	0	0.00	0.00, 0.43	0	0.00	0.00, 0.43	0.00	−0.43, 0.43
		60	20	9	0.45	0.26, 0.66	7	0.35	0.18, 0.57	0.10	−0.19, 0.37
		200	5	5	1.00	0.57, 1.00	5	1.00	0.57, 1.00	0.00	−0.43, 0.43

a N = Number of test portions.

b x = Number of positive test portions.

c POD_C_ = Candidate method presumptive positive outcomes confirmed positive using the reference method procedure.

d POD_R_ = Reference method confirmed positive outcomes divided by the total number of trials.

e dPOD_C_ = Difference between the candidate method and reference method POD values.

f 95% CI = If the confidence interval of a dPOD does not contain zero, then the difference is statistically significant at the 5% level.

g CWD = University of Vermont, Burlington, VT.

h ATCC = American Type Culture Collection, Manassas, VA.

i N/A = Not applicable.

**Table 7.** PhageDx *Listeria* results: presumptive vs confirmed using the PhageDx *Listeria* confirmation procedure

Matrix	Strain	CFU/test area	N^a^	PhageDx *Listeria* presumptive			PhageDx *Listeria* confirmed			${\text{dPOD}}_{\text{CP}}^{e}$	95% CI^f^
				x^b^	${\text{POD}}_{\text{CP}}^{c}$	95% CI	x	${\text{POD}}_{\text{CC}}^{d}$	95% CI		
Stainless steel (4 × 4”)	*L. monocytogenes* 4a (CWD 1554)/E. faecalis ATCC 29212^g^^,h^	N/A^i^	5	0	0.00	0.00, 0.43	0	0.00	0.00, 0.43	0.00	−0.47, 0.47
		48 & 510	20	8	0.40	0.22, 0.61	8	0.40	0.22, 0.61	0.00	−0.13, 0.13
		160 & 1800	5	5	1.00	0.57, 1.00	5	1.00	0.57, 1.00	0.00	−0.47, 0.47
Ceramic (4 × 4”)	L. innocua (ATCC 43547 6 b)	N/A	5	0	0.00	0.00, 0.43	0	0.00	0.00, 0.43	0.00	−0.47, 0.47
		60	20	9	0.45	0.26, 0.66	9	0.45	0.26, 0.66	0.00	−0.13, 0.13
		200	5	5	1.00	0.57, 1.00	5	1.00	0.57, 1.00	0.00	−0.47, 0.47

a N = Number of test portions.

b x = Number of positive test portions.

c POD_CP_ = Candidate method presumptive positive outcomes divided by the total number of trials.

d POD_CC_ = Candidate method confirmed positive outcomes (confirmed using the PhageDx Listeria recommended procedure) divided by the total number of trials.

e dPOD_CP_ = Difference between the candidate method presumptive result and candidate method confirmed result POD values.

f 95% CI = If the confidence interval of a dPOD does not contain zero, then the difference is statistically significant at the 5% level.

g CWD = University of Vermont, Burlington, VT.

h ATCC = American Type Culture Collection, Manassas, VA.

i N/A = Not applicable.

**Table 8.** Method comparison results: PhageDx *Listeria* vs BAM Ch. 10

Matrix	Strain	CFU/test area	N^a^	PhageDx *Listeria* results			BAM Ch. 10			${\text{dPOD}}_{\text{C}}^{e}$	95% CI^f^
				x^b^	${\text{POD}}_{\text{C}}^{c}$	95% CI	x	${\text{POD}}_{\text{R}}^{d}$	95% CI		
Stainless steel (4 × 4”)	*L. monocytogenes* 4a (CWD 1554)/E. faecalis ATCC 29212^g^^,h^	N/A^i^	5	0	0.00	0.00, 0.43	0	0.00	0.00, 0.43	0.00	−0.47, 0.47
		48 & 510	20	8	0.40	0.22, 0.61	8	0.40	0.22, 0.61	0.00	−0.13, 0.13
		160 & 1800	5	5	1.00	0.57, 1.00	5	1.00	0.57, 1.00	0.00	−0.47, 0.47
Ceramic (4 × 4”)	*L. innocua* (ATCC 43547 6 b)	N/A	5	0	0.00	0.00, 0.43	0	0.00	0.00, 0.43	0.00	−0.43, 0.43
		60	20	9	0.45	0.26, 0.66	7	0.35	0.18, 0.57	0.10	−0.19, 0.37
		200	5	5	1.00	0.57, 1.00	5	1.00	0.57, 1.00	0.00	−0.43, 0.43

a N = Number of test portions.

b x = Number of positive test portions.

c POD_C_ = Candidate method presumptive positive outcomes confirmed positive using the PhageDx *Listeria* recommended procedure.

d POD_R_ = Reference method confirmed positive outcomes divided by the total number of trials.

e dPOD_C_ = Difference between the candidate method and reference method POD values.

f 95% CI = If the confidence interval of a dPOD does not contain zero, then the difference is statistically significant at the 5% level.

g CWD = University of Vermont, Burlington, VT.

h ATCC = American Type Culture Collection, Manassas, VA.

i N/A = Not applicable.

## Discussion

The results of this validation study show that the PhageDx *Listeria* Assay is an effective alternative to the FDA/BAM Chapter 10 for the detection of *Listeria* on stainless steel and ceramic surfaces.

In inclusivity and exclusivity testing, the method was shown to be specific for *Listeria*, correctly identifying 58/61 *Listeria* target strains including all 34 *L. monocytogenes* strains tested. The PhageDx *Listeria* Assay was unable to detect three strains within the inclusivity panel, one strain each of *L. innocua* (ATCC BAA-680), *L. seeligeri* (ATCC 51335), and *L. welshimeri* (ATCC 43551)*.* It is unclear exactly why these strains failed to generate a positive result since the PhageDx *Listeria* Assay was able to detect other members of the species. One possible explanation is that these strains do not have the receptor(s) or the optimal receptors required for efficient recognition by the phage (9). Possessing a receptor that was sufficiently similar to *Listeria* also may explain why *Lactobacillus plantarum* generated a false positive result. Another possibility for a false negative may be that the strain has a mechanism that prevents the phage from producing luciferase. These mechanisms may include, but are not limited to, preventing phage absorption, preventing phage DNA entry, or the cutting of phage nucleic acids (10).

The recombinant phage can be produced consistently and is stable for 6 months when stored appropriately. Robustness testing of the PhageDx *Listeria* Assay indicated that the method works well when the assay parameters (infection time, recombinant phage concentration, and substrate amount) were varied from the stated protocol.

Independent laboratory testing demonstrated that the PhageDx *Listeria* Assay was able to detect *Listeria* at low levels and in the presence of competing bacteria on stainless steel and ceramic surfaces. These studies also demonstrated that the performance of the PhageDx *Listeria* Assay was not statistically different from that of FDA/BAM Chapter 10 for environmental surfaces. Additionally, the PhageDx confirmation procedure was shown to produce results identical to the FDA/BAM Chapter 10 reference method confirmation procedure.

The PhageDx *Listeria* Assay has a number of advantages over the FDA/BAM Chapter 10 reference method. In addition to being a specific assay, the results are easy to interpret as an RLU endpoint is used to determine the outcome of the assay. Another advantage is that PhageDx provides a presumptive positive result in as little as 24.5 h compared to 72+ h in the case of the FDA/BAM Chapter 10 reference method. PhageDx is also a simple test that involves only five basic steps: enrichment, sampling, infection, substrate addition, and signal readout. Finally, PhageDx Assay is a rapid method that offers a considerable time savings compared to the FDA/BAM Chapter 10 reference method.

## Conclusion

Results of this validation study support the claim that the PhageDx *Listeria* Assay is a specific, sensitive, fast, and simple method for the detection of *Listeria* on stainless steel and ceramic surfaces and is statistically comparable to the FDA/BAM Chapter 10 reference method. By using a luciferase-expressing recombinant bacteriophage, the PhageDx Assay was able to detect a positive sample after 20 h enrichment and a 4 h infection for environmental surfaces. The PhageDx *Listeria* Assay thus offers a significantly shorter time to results compared with the FDA/BAM Chapter 10 reference method.

## Submitting Company

Laboratory Corporation of America^®^

531 South Spring Street

Burlington, NC 27215

## Independent Laboratory

**Kateland Koch, Benjamin Bastin, M. Joseph Benzinger, Jr., and James Agin**

Q Laboratories, Inc.

1930 Radcliff Drive

Cincinnati, OH 45204, USA

## Maria Cristina Fernandez

Maimonides University

Hidalgo 775 (esq. Felipe Vallese)

C1405BCK Buenos Aires, Argentina

## Michael Brodsky

Brodsky Consultants

73 Donnamora Crescent

Thornhill

Ontario L3T 4K6, Canada
